# Characterization and phylogenetic analysis of the complete mitochondrial genome of *Saccharomycopsis fibuligera* (lindner) Klocker 1907 (saccharomycetales: saccharomycopsidaceae)

**DOI:** 10.1080/23802359.2024.2364756

**Published:** 2024-06-14

**Authors:** Yue Deng, Guangjiu Chen, Xuedong Bao, Jie He

**Affiliations:** Luzhou Vocational and Technical College, Luzhou, Sichuan, China

**Keywords:** Mitochondrial genome, brewing yeast, evolution, phylogeny

## Abstract

*Saccharomycopsis fibuligera* (Lindner) Klocker 1907 is frequently employed in the fermentation of metabolites such as citric acid, ethanol, mannitol, and pyruvate. Its heat tolerance and alcohol-producing capabilities during fermentation make it a desirable option for bread and wine production. To date, the mitochondrial genome of *S. fibuligera* has not been sequenced. In the present study, we obtained the full mitochondrial genome of *S. fibuligera*, which is 57,302 bp long and has a GC content of 24.40%. This genome contained 14 core protein-coding genes, 3 independent ORFs, 21 intronic ORFs, 25 tRNAs, and 2 rRNA genes. By utilizing the Bayesian inference phylogenetic method, we constructed phylogenetic trees for 24 Saccharomycotina fungi, which indicated that *S. fibuligera* is closely related to *S. capsularis*.

## 1. Introduction

*Saccharomycopsis fibuligera* (Lindner) Klocker 1907 is an aerobic yeast species that has garnered attention for its ability to grow on a variety of carbon sources, including glucose, xylose, and glycerol (Chi et al. 2009; Moon et al. 2021). Its tolerance to high osmolarity environments makes it an ideal candidate for biotechnological applications such as the production of biofuels (Favaro et al. 2015). Moreover, *S. fibuligera* has been widely used in the fermentation of metabolites such as citric acid, ethanol, mannitol, and pyruvate (Su et al. 2020; Yang et al. 2021). Furthermore, its ability to tolerate high temperatures and form alcohols during fermentation makes it a suitable choice for bread and wine making (Lee et al. 2018; Farh et al. 2021; Methner et al. 2022). Consequently, *S. fibuligera* is increasingly recognized for its diverse adaptability and unique characteristics.

The mitochondrial genome of eukaryotes is indispensable for the regulation of growth and development, sustaining homeostasis and enabling the cell to react to the environment (Ernster and Schatz 1981; McBride et al. 2006; Murphy 2009). It has been suggested that the mitochondrial genome is a useful resource for examining fungal phylogeny (Xu and Wang 2015; Li et al. 2022a, 2022b, 2023a; Gao et al. 2024). To date, two mitochondrial genomes from the *Saccharomycopsis* genus have been sequenced (Wolters et al. 2023). However, the mitochondrial genome characteristics of *S. fibuligera* have not been revealed. In the present study, we obtained the complete mitochondrial genome of *S. fibuligera* for the first time through next-generation sequencing technology, which has improved our understanding of the characteristics and evolution of the mitochondrial genome in the *Saccharomycopsis* genus.

## 2. Materials and methods

### 2.1. Sample collection

**Figure 1.**
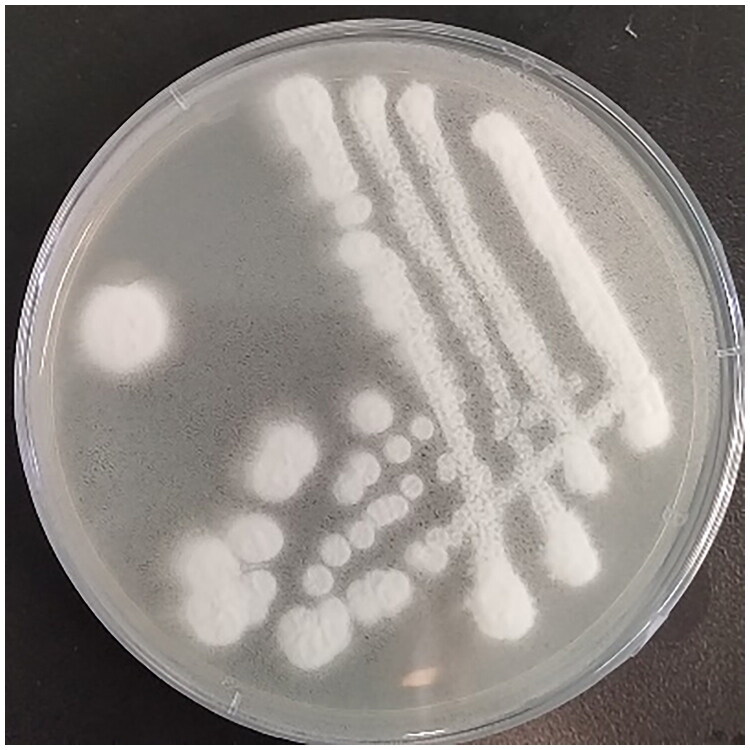
Isolation of the brewing yeast *Saccharomycopsis fibuligera*. This fungus was cultured on PDA media (200 g/L potato, 20 g/L glucose, and 20 g/L agar) at 28 °C for 3 days. A photo of the species was taken by Yue Deng.

In 2023, a specimen of *S. fibuligera* was isolated from a wine fermentation system in Luzhou (E 105.40°, N 28.91°), Sichuan, China. Morphological and ITS rRNA sequencing were used to identify the specimens, which were then deposited at the Culture Collection Center of Luzhou Vocational and Technical College (contact person: Yue Deng; email: 157317724@qq.com) with voucher number Sfib1 (Figure 1).

### 2.2. Mitochondrial genome assembly and annotation

For DNA extraction from *S. fibuligera*, a fungal DNA extraction kit from Omega Bio-Tek (Norcross, GA, USA) was used. The NEBNext® Ultra™ II DNA Library Prep Kit (NEB, Beijing, China) was then used for sequencing library preparation according to the manufacturer’s instructions. Subsequently, the Illumina HiSeq 2500 Platform (Illumina, San Diego, CA, USA) was used for whole-genome sequencing. To guarantee the accuracy of the data, ngsShoRT (Chen et al. 2014) was used to filter out low-quality sequences, and AdapterRemoval v2 (Schubert et al. 2016) was used to remove adapter reads. The mitochondrial genome of *S. fibuligera* was de novo assembled using version 4.3.3 of NOVOPlasty, with a k-mer size of 31 (Dierckxsens et al. 2017). The mitochondrial genome was annotated in accordance with previously described methods (Li et al. 2019, 2020b, 2023b), which involved the use of the MFannot tool (Lang et al. 2023) and MITOS (Bernt et al. 2013). By using the NCBI Open Reading Frame Finder, we can predict or modify protein-coding genes (PCGs) or open reading frames (ORFs) that are longer than 100 amino acids (Wu et al. 2017). Annotation of the functions of PCGs or ORFs was accomplished through BLASTP searches against the NCBI nonredundant protein sequence database (Bleasby and Wootton 1990). Exon and intron boundaries of PCGs were accurately identified with the help of exonerate version 2.2 (Slater and Birney 2005). Using tRNAscan-SE v1.3.1, we confirmed the presence of tRNA genes in the *S. fibuligera* mitochondrial genome (Lowe and Chan 2016). OGDraw v1.2 was used to generate a graphical representation of the mitochondrial genome (Lohse et al. 2013). The structures of intron-containing genes were visualized using the PMGmap online web (http://www.1kmpg.cn/pmgmap, Supplementary Figure 1) (Zhang et al. 2024).

### 2.3. Phylogenetic analysis

The phylogenetic tree was constructed using methods described previously (Li et al. 2020a, 2021, 2022c). Using MAFFT v7.037 software, we initiated the process by aligning individual mitochondrial genes (excluding intron regions) (Katoh et al. 2019). Utilizing SequenceMatrix v1.7.8, we connected the aligned mitochondrial genes to form a single, unified mitochondrial dataset (*atp6, atp8, atp9, cob, cox1, cox2, cox3, nad1, nad2, nad3, nad4, nad4L, nad5*, and *nad6*) (Vaidya et al. 2011). To detect any phylogenetic discrepancies between distinct mitochondrial genes, an initial partition homogeneity test was performed. PartitionFinder 2.1.1 was utilized to pinpoint the most suitable models of partitioning and evolutionary processes for the merged mitochondrial dataset (Lanfear et al. 2017). MrBayes v3.2.6 was utilized to construct phylogenetic trees by applying Bayesian inference (Ronquist et al. 2012).

## 3. Results

**Figure 2.**
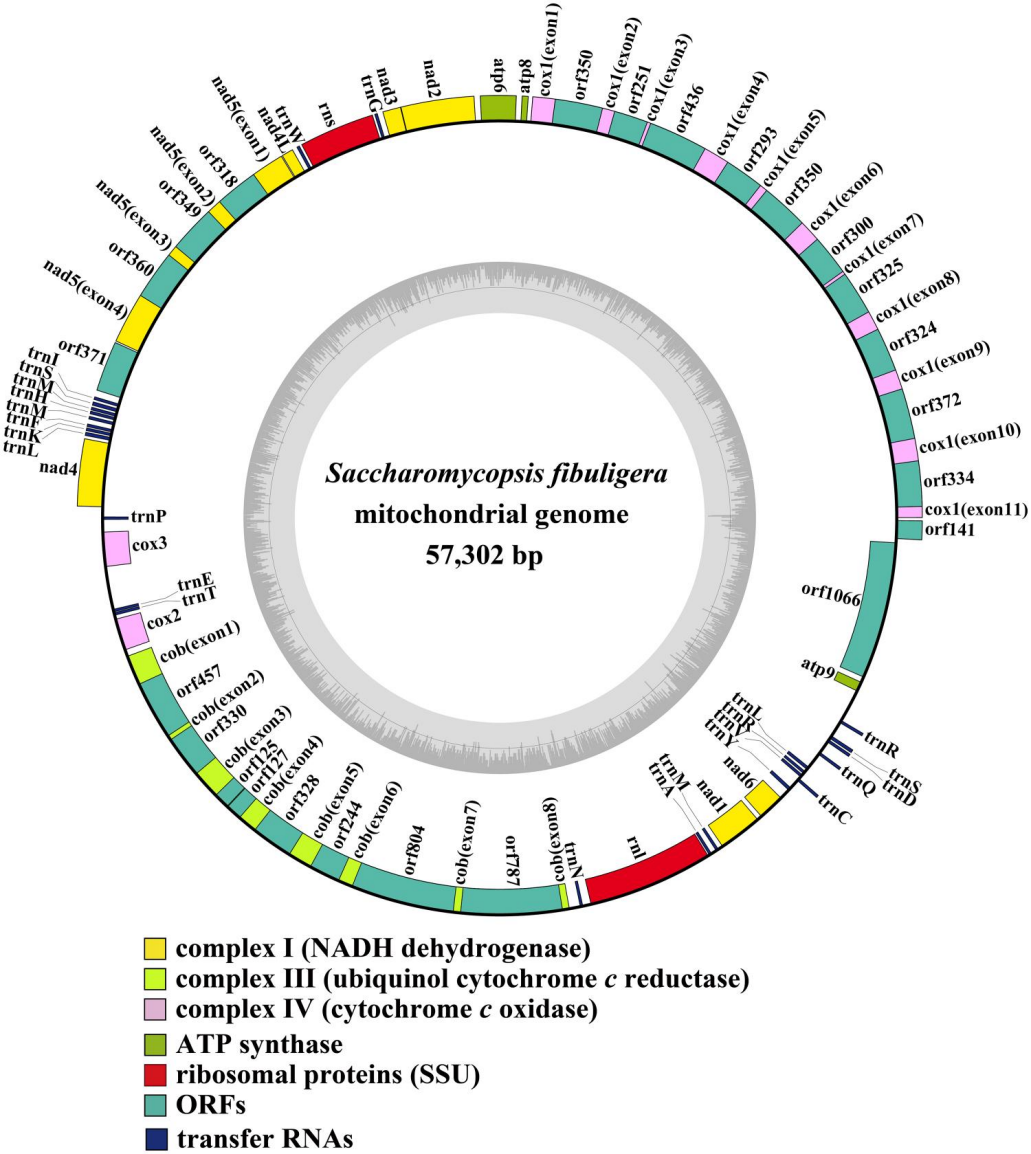
Circular mitochondrial genome map of *Saccharomycopsis fibuligera.* Colored blocks outside each ring indicate that the genes are on the direct strand, while colored blocks within the ring indicate that the genes are located on the reverse strand. The inner grayscale bar graph shows the GC content of the mitochondrial sequences. The circle inside the GC content graph marks the 50% threshold.

**Figure 3.**
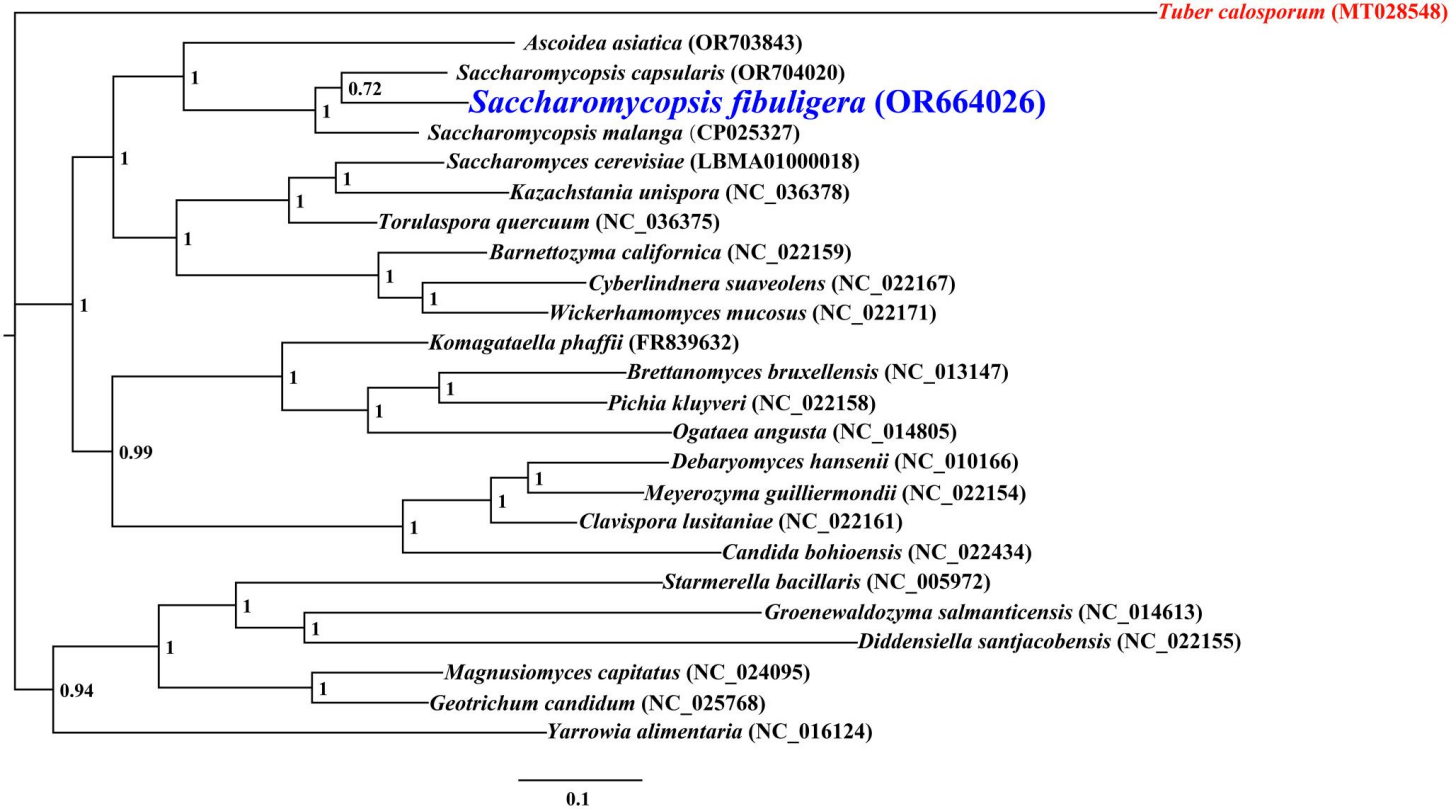
Bayesian inference (BI) tree generated using 14 concatenated mitochondrial protein-coding genes (*atp6*, *atp8*, *atp9, cob, cox1, cox2, cox3, nad1, nad2, nad3, nad4, nad4L, nad5,* and *nad6*) from *Saccharomycopsis fibuligera* and 23 other fungal species from saccharomycotina. The number next to the evolutionary branch node is the bayesian posterior probability (BPP). *Tuber calosporum* was used as the outgroup (Li et al. 2020c). the accession number information of the sequence is as follows: *Diddensiella santjacobensis* (NC_022155), *kazachstania unispora* (NC_036378) (Xiao et al. 2017), *ogataea angusta* (NC_014805) (Eldarov et al. 2011), *yarrowia alimentaria* (NC_016124) (Gaillardin et al. 2012), *pichia kluyveri* (NC_022158), *Magnusiomyces capitatus* (NC_024095) (Lang et al. 2014), *Geotrichum candidum* (NC_025768) (Morel et al. 2015), *tuber calosporum* (MT028548) (Li et al. 2020c), *saccharomycopsis capsularis* (OR704020) (Wolters et al. 2023), *saccharomycopsis malanga* (CP025327), *ascoidea asiatica* (OR703843) (Wolters et al. 2023), *Saccharomycopsis fibuligera* (OR664026), *barnettozyma californica* (NC_022159), *Saccharomyces cerevisiae* (LBMA01000018) (McIlwain et al. 2016), *brettanomyces bruxellensis* (NC_013147) (Procházka et al. 2010), *groenewaldozyma salmanticensis* (NC_014613) (Valach et al. 2011), *komagataella phaffii* (FR839632) (Küberl et al. 2011), *cyberlindnera suaveolens* (NC_022167), *torulaspora quercuum* (NC_036375) (Xiao et al. 2017), *Clavispora lusitaniae* (NC_022161), *candida bohioensis* (NC_022434), *Meyerozyma guilliermondii* (NC_022154), *wickerhamomyces mucosus* (NC_022171), *starmerella bacillaris* (NC_005972) (Pramateftaki et al. 2008), and *Debaryomyces hansenii* (NC_010166) (Sacerdot et al. 2008).

The average depth of the coverage-depth map was 7934.8× (Supplementary Figure 2), and the mitochondrial genome was 57,302 bp long with a GC content of 24.40%. Analysis of the *S. fibuligera* mitochondrial genome revealed 38 open-reading frames, which included 14 core PCGs (*cox1, cox2, cox3, atp6, atp8, atp9, cob, nad1, nad2, nad3, nad4, nad4L, nad5,* and *nad6*), 3 free-standing ORFs, and 21 intronic ORFs (Figure 2). The *S. fibuligera* mitochondrial genome was found to contain 20 introns, with 12 belonging to Group IB, 2 to Group IA, 2 to Group ID, 2 to Group II, 1 to Group I (derived), and 1 to unknown types. Intronic ORFs encoding LAGLIDADG-homing endonucleases or GIY-YIG-homing endonucleases were present in some of the introns. The mitochondrial genome of *S. fibuligera* was found to contain two ribosomal RNA genes, the small subunit (*rns*) and the large subunit (*rnl*), as well as 25 transfer RNA genes. Phylogenetic analysis demonstrated that *S. fibuligera* is closely related to *S. capsularis*, as depicted in Figure 3.

## 4. Discussion and conclusion

By utilizing the mitochondrial genome, we can gain a more comprehensive understanding of the phylogenetic relationships between species (Zhang et al. 2020; Ren et al. 2021; Zhang et al. 2022; 2023). The absence of a mitochondrial reference genome for *S. fibuligera* impedes the application of the mitochondrial genome for classifying and investigating the phylogenetic relationships of Saccharomycopsidaceae fungi. In this study, we acquired the full mitochondrial genome of *S. fibuligera*. It was 57,302 bp in length, with a GC content of 24.40%. This genome included 14 core protein-coding genes (PCGs), 3 independent ORFs, 21 intronic ORFs, 25 tRNAs, and 2 rRNA genes. The *S. fibuligera* mitochondrial genome is three times smaller than the mitochondrial genome of *S. malanga*. By employing the BI phylogenetic inference method, we were able to construct phylogenetic trees for 24 Saccharomycotina fungi, with strong support for major clades (Shen et al. 2018; Groenewald et al. 2023); this demonstrated that *S. fibuligera* has a close relationship with *S. capsularis*. This study provides valuable information that is indispensable for the identification and recognition of *Saccharomycopsis* species, thus increasing our understanding of mitochondrial evolution and the varieties of Saccharomycopsidaceae fungi.

## Ethics statement

The study did not involve humans or animals. In this study, samples were collected by the authors without ethical approval or permission.

## Data Availability

The genome sequence data that support the findings of this study are openly available in the NCBI GenBank at https://www.ncbi.nlm.nih.gov/under accession no. OR664026. The associated BioProject, SRA, and Bio-Sample numbers are PRJNA1025866, SRR26320845 and SAMN37723664, respectively.
